# Exploring correlates of high psychiatric inpatient utilization in Switzerland: a descriptive and machine learning analysis

**DOI:** 10.1186/s12888-024-06388-6

**Published:** 2024-12-23

**Authors:** Mariela E. Jaffé, Stefan Weinmann, Andrea H. Meyer, Helen Stepulovs, Regula Luethi, Stefan Borgwardt, Roselind Lieb, Undine E. Lang, Christian G. Huber, Julian Moeller

**Affiliations:** 1 University Psychiatric Clinics (UPK), University of Basel, Basel, Switzerland; 2 Division of Clinical Psychology and Epidemiology, Department of Psychology, University of Basel, Basel, Switzerland; 3 University Hospital Zurich, Zurich, Switzerland; 4 Department of Psychiatry and Psychotherapy, University of Luebeck, Luebeck, Germany; 5 Center of Social Psychology, University of Basel, Basel, Switzerland

**Keywords:** High utilization, Psychiatric inpatient services, Re-hospitalisation, Revolving door, Severe mental illness

## Abstract

**Background:**

This study investigated socio-demographic, psychiatric, and psychological characteristics of patients with high versus low utilization of psychiatric inpatient services. Our objective was to better understand the utilization pattern and to contribute to improving psychiatric care.

**Methods:**

One-hundred and twenty inpatients of the University Psychiatric Clinics (UPK) Basel, Switzerland, participated in this cross-sectional study. All patients were interviewed using different clinical scales. As target variables we investigated the number of days of psychiatric inpatient treatment within a 30-month period.

**Results:**

Despite including multiple relevant patient variables and using elaborate statistical models (classic univariate und multiple regression, LASSO regression, and non-linear random forest models), the selected variables explained only a small percentage of variance in the number of days of psychiatric inpatient treatment with cross-validated *R* ^*2*^ values ranging from 0.16 to 0.22. The number of unmet needs of patients turned out to be a meaningful and hence potentially clinically relevant correlate of the number of days of psychiatric inpatient treatment in each of the applied statistical models.

**Conclusions:**

High utilization behavior remains a complex phenomenon, which can only partly be explained by psychiatric, psychological, or social/demographic characteristics. Self-reported unmet patient needs seems to be a promising variable which may be targeted by further research in order to potentially reduce unnecessary hospitalizations or develop better tailored psychiatric treatments.

## Background

In a perfect world, people with mental health problems can turn to health professionals in their local communities to receive help and support whenever indicated. If the situation is more severe, outpatient support may not suffice and inpatient psychiatric treatment could be required (cf., stepped care and collaborative care models; e.g., [1–3]). The simplified objective of a stay in a psychiatric hospital is the provision of effective multidisciplinary treatment that supports patients in their recovery and allows reaching the highest quality of life. Depending on the respective guidelines, such a treatment may entail psychotherapy, medication, psychiatric nursing care, and many other options that the respective hospital has to offer and the patients like and need. Once the individual objective is reached, the patients leave the inpatient setting and may receive further support within their local communities. Ideally, the main part of mental health care takes place in the outpatient setting (cf., the outpatient before inpatient policy in Switzerland, [4], see also [5], and a perspective on associated stigma by [6]). Inpatient stays would tend to be short and a more exceptional case (e.g., starting with least restrictive and intensive care and reserve more intensive treatment for “people who do not benefit from first-line treatments”, as described in the stepped care approach by [1, 7], see also “balanced care” as described in [8]). This, however, is not always the case. Many patients need to be readmitted to psychiatric hospitals and experience multiple stays throughout a certain period or their lifetime [9]. On the one hand, this may indicate that a specific group of patients rely on comparatively frequent inpatient treatment to obtain a sufficient treatment outcome in certain life periods. On the other hand, this may also point to the possibility that adequate treatment and care by multidisciplinary teams outside of the hospital are not accessible in mental health crises or are not sufficiently tailored to this patient group’s specific characteristics and needs.

### (High) utilization of psychiatric inpatient treatment

To better understand the utilization of psychiatric inpatient treatment, previous research systematically focused on the number of days specific patients spent in the hospital and relevant correlates which might be associated with the amount of time spent there. Researchers identified that a small percentage of patients utilize a large number of inpatient treatment days provided by hospitals. This pattern has been described as *high utilization* (HU) of psychiatric care and the group of patients that require proportionally larger numbers inpatient treatment days as *high utilizers* [9–11]. Other terms used in the literature are heavy or frequent user (see, e.g., [12]) or revolving door patients [13].

Previous research investigated the high utilization phenomenon and highlighted differences between patients with a high versus low utilization profile (see, e.g., [14]). Regarding gender, previous studies reported inconclusive results, as some studies indicate a higher proportion of male patients (e.g., [15]), whereas others point to a higher proportion of female patients with a high utilization profile (e.g., [16]). Patients with high utilization tend to be younger [17, 18]. Other work indicated that high versus low utilization was more likely associated with a diagnosis of schizophrenia, schizoaffective disorder, or bipolar disorder [9, 16, 19–21] as well as comorbid substance use/abuse or personality disorder [14, 16, 20–22]. Symptomatic burden as well as lower integration into the local social and health services network were associated with a higher utilization pattern [11].

The use of varying definitions of high-utilization, however, – which could result in the same behavior only sometimes being classified as high utilization – limits the current knowledge in this research field and consequently the development of standardized targeted interventions for patients with a high utilization profile of psychiatric inpatient treatment (e.g., [14]).

While the number of studies examining the high utilizer phenomenon in psychiatry has increased in recent decades [23], there are comparatively few such studies in Switzerland (but see, e.g., [9, 10, 24]). This is a research gap, as the underlying correlates of high utilization may vary across health care systems (depending on, e.g., the number of inpatient beds, number of psychiatrists, psychiatric nurses, psychologists or social workers in relation to the population requiring mental health care; cf., [25, 26]) and may not be generalizable to other patient groups. Describing high utilizer patients in a respective health care systems is important to tailor evidence-based interventions to their specific characteristics and needs as well as to identify incentives and methods to change this.

In this cross-sectional study, we therefore report data from a hospital with a catchment area in a German-speaking part of Northern Switzerland, thereby focusing on the health care system of Switzerland. The aim was to provide a detailed picture of the characteristics and needs of patients retrospectively classified with a high versus low utilization pattern of psychiatric inpatient treatment. We explore socio-demographic (e.g., gender, age, living situation), psychiatric (e.g., the current primary diagnosis, comorbidities) and psychological (e.g., attitudes towards medication, the extent of (un)fulfilment regarding patients’ needs) variables, which have been associated with higher utilization of psychiatric inpatient treatment in previous international studies. First, we describe patients with and without a high utilization profile ([10], cf., [11]). Secondly, we examine potential correlates of utilization of psychiatric inpatient treatment with inferential statistics – including machine-learning approaches. This study therefore provides a precise description of the respective patient sample as well as an analysis of correlates of high utilization of psychiatric inpatient treatment in Northern Switzerland. We intended to contribute to a broader understanding of the complex nature of this health care phenomenon in Switzerland and explore possible starting points for future research and practice in order to meet the needs of patients with multiple or long inpatient stays.

## Methods

This cross-sectional study was conducted at the Clinic for Adults of the University Psychiatric Clinics (UPK) Basel, University of Basel, Switzerland. The clinic is a large psychiatric university hospital that provides both inpatient and outpatient mental health services to a population of approximately 200 000 individuals in the surrounding area (cf., [27]). At the time of the study, the clinic had approximately 211 beds available for inpatient treatment. Additionally, the clinic offers day clinic services and outpatient care for all patient groups. Outpatient mental health care, however, is also provided by other institution’s outpatient clinics and by an independent network of psychiatrists and psychologists working in practices (see also [28]).

Participating patients were treated in four acute psychiatric inpatient wards of the former centre for diagnostics and crisis intervention. At the time of the study, the wards specialized in the treatment of patients with acute psychoses, depressions and/or personality disorders, with or without compulsory admissions. Ethics approval was obtained from the Approval Committee “Ethics Committee Northwest and Central Switzerland (EKNZ)” under protocol number EKNZ BASEC 2016–00407 (Clinical trial number: not applicable). All participants provided informed consent and the study was carried out in compliance with the Declaration of Helsinki. Participants were recruited between August 2016 and May 2017.

### Participants

Patients who had been admitted to inpatient psychiatric treatment in one of the four psychiatric wards, who were between 18–65 years old, and had a current primary diagnosis of a mental or behavioral disorder according to ICD-10 in the domains F2X (schizophrenia, schizotypal, and delusional disorders), F3X (mood / affective disorders), F4X (neurotic, stress related, and somatoform disorders), or F6X (disorders of personality and behavior in adult persons) were included in the study. Patients within psychiatric day hospital treatment, patients of forensic facilities, patients with dementia or organic mental disorders (F0X) or a primary diagnosis of substance abuse (F1X), homeless patients, and/or patients with insufficient language skills or who were not able to provide informed consent were not included.

### Procedure

Within one week of admission to psychiatric inpatient treatment, all eligible patients were contacted via the physician in charge. Patients were then invited to participate in the study. In the case of patients with acute psychotic symptoms, the possibility of study participation was regularly evaluated over the course of the inpatient stay and patients were invited to participate in the study at a later time, if indicated. If patients were willing to participate, a psychologist arranged a meeting in the ward and informed them about the study. The psychologist interviewed patients who provided informed consent using a semi-structured psychiatric interview that lasted between 45 and 60 min; the interview could also be divided into two sessions if the patient preferred a break. Before the interview was conducted, background information on socio-demographic details (age, gender, nationality, marital status, occupational and educational status), psychiatric (current diagnosis of mental disorder, comorbid mental disorders, somatic disorders) and treatment-related variables (frequency and duration of hospital inpatient admissions) were collected via the electronic patient files, if available, and inserted into the questionnaire. This procedure allowed us to collect data on the frequency or duration of hospital inpatient admissions during the last 30 months that might have been difficult to recall in a personal interview. However, if patients reported having utilized inpatient treatment outside of UPK Basel during the interview, this information was complemented and integrated for analyses. Furthermore, additional information was assessed (see below). Patients assigned to the four psychiatric wards several times during the recruitment period of the study were eligible to participate only once.

### Materials

The assessments were done by a psychologist (trained rater) using a semi-structured interview containing the following scales on the utilization of treatment, socio-demographic, psychiatric, and psychological information. The reliability coefficients reported below refer to the sample studied in this manuscript.

#### Client sociodemographic and service receipt inventory (CSSRI, [29, 30])

The respondents’ use of inpatient and community-based care was recorded with the Client Sociodemographic and Service Receipt Inventory. The CSSRI is a questionnaire developed by Chisholm and colleagues [29], translated to German by Roick and colleagues [30], and adapted to Swiss conditions by the research and development department of the psychiatric hospital of the University of Basel. The inventory collects retrospective information on the interviewee’s utilization of health and social care services, accommodation and living situations, income, employment, and receipt of benefits, with a focus on the 6-month period preceding the assessment. Exemplary items regarding utilization of health care services focus on inpatient stays in psychiatric clinics, day clinics, and outpatient appointments.

Within this inventory we assessed utilization of inpatient treatment during the last 30 months prior to study participation. We used information from electronic patient files for inpatient stays at UPK Basel and self-reports for stays in other hospitals. For descriptive analyses, we categorized utilization into high and non-high utilization of inpatient psychiatric treatment. Based on previous research, utilization was categorized as high (HU) if a patient spent ≥ 180 days and/or at least 3 stays of at least 3 days in psychiatric inpatient treatment during the last 30 months ([10], cf., [11]). If this threshold was not met, utilization was categorized as non-high (NHU). For inferential analyses, we created the continuous target variable number of treatment days by summarizing the total number of days spent in psychiatric inpatient clinics during the last 30 months (assessed in retrospect, meaning up to the current inpatient stay).

#### Brief psychiatric rating scale (BPRS, [31])

The interviewer used the BPRS-18 to rate the current mental health status of the patients. The BPRS-scale is a general psychiatric rating scale containing 18 ordered categories of symptoms of mental illness, which are rated on a 7-point Likert scale (1 = *not present*, 7 = *extremely severe*). An overall pathology score is obtained by summing the ratings across the 18 items. The scale is widely used in clinical research [32]. It was developed in its original English version as a 16-item version [33] and has subsequently been replaced by an 18-item version [31]. We used the German version of the scale [34]. Cronbach’s alpha for the BPRS scale is 0.69. For analysis, we focused on five subscales (ECDEU, cf., [32]), namely anxiety depression (Cronbach’s alpha = 0.62), anergia (Cronbach’s alpha = 0.58), thought disturbance (Cronbach’s alpha = 0.65), hostile suspiciousness (Cronbach’s alpha = 0.52), and activation (Cronbach’s alpha = 0.63).

#### Global assessment of functioning scale (GAF, [35])

Current global functioning was rated using the GAF scale. The scale serves as a clinician-rated, reliable, and valid instrument to measure axis V symptom severity or psychological, social, and occupational functioning ( [36], cf., [37]). Ratings are located on a continuum ranging from 1 to 100, with 100 indicating the highest functioning level. We used the German translation of the GAF scale by Saß and colleagues [38].

#### Camberwell assessment of need short appraisal (CANSAS, [39, 40])

Participants completed the CANSAS to assess the current status of their needs across 22 domains (e.g., living situation, diet, and other) within the last four weeks. For each area of need a self-reported rating is provided to indicate whether this topic is perceived as 0 = *not problematic*, 1 = *not or only somewhat problematic as support is provided*, 2 = *a serious problem*, or 9 = *unknown*. A count score can then be calculated for each patient to indicate the number of met, unmet, and total needs. CANSAS has been recognized as a valid and reliable instrument for assessing the needs of people with severe mental illness [39, 41]. We used the German translation provided by Kilian and colleagues [41].

#### Drug attitude inventory (DAI-10, [42, 43])

Participants also completed a self-report questionnaire consisting of 10 statements measuring the perceived effects and benefits of psychopharmacologic medication in general. Patients could indicate agreement or disagreement with each item. Higher scores indicate a more positive attitude towards and a more positive experience regarding medication. A German translation of the inventory was used in this study. Cronbach’s alpha for the DAI-10 scale is 0.69. In line with the work by Kim and colleagues [44], we computed scores for the three subscales, indicating a subjective positive response (Cronbach’s alpha = 0.68), subjective negative response (Cronbach’s alpha = 0.59), and attitude to medication (Cronbach’s alpha = 0.43).

#### Sociodemographic questions

We interviewed participants in regards to their basic sociodemographic information using a short self-developed questionnaire. An English translation of the questionnaire can be found in Appendix A.

Additional information on the study materials and the data collection can be found in Appendix B.

### Design, data preparation and statistical analyses

In this cross-sectional study, we analyzed associations between two sets of variables: patients’ utilization behavior during the last 30 months operationalized by the number of treatment days, on the one hand, and their current socio-demographic, psychiatric, and psychological variables on the other hand. For the descriptive part of the study, we report an account of the overall characteristics of the study participants and separately for patients with a treatment history categorized as HU or NHU. Categorization based on continuous outcomes is, however, associated with a loss of information. For inferential statistics, we therefore look at the continuous target variable number of treatment days. Based on the existing literature and research while keeping power and feasibility concerns in mind, we selected a priori 23 potential correlates for the regression analyses. As socio-demographic correlates, we selected gender (coded as 0 = *male* and 1 = *female participant*), age, nationality (0 = *Swiss*, 1 = *other*), family status (0 = *no relationship*, 1 = *in a relationship*), educational level (0 = *low*, 1 = *medium*, 2 = *high*), working situation (0 = *regular labour market*, 1 = *protected labour market*, 2 = *no job*), living situation (0 = *assisted living*, 1 = *other*), and receipt of disability pension (0 = *no*, 1 = *yes*). As psychiatric correlates, we selected the presence of an F2X diagnosis (0 = *other*, 1 = *F2X diagnosis*), F30/F31 diagnosis (0 = *no*, 1 = *yes*), F32/F33 diagnosis (0 = *no*, 1 = *yes*), the presence of a diagnosis of a psychiatric comorbidity (0 = *no*, 1 = *yes*), the presence of addiction as a diagnosed comorbidity (0 = *no*, 1 = *yes*), the BRPS subscales anxiety-depression, anergia, thought disturbance, activation, and hostile suspiciousness, as well as the GAF-score. As psychological and need-related correlates, we selected the three DAI subscales subjective positive response, subjective negative response, and attitude to medication. Furthermore, we computed a count score of the needs that have been identified as unmet (2 = *a serious problem*).

For the inferential analyses, we chose a four-step approach. First, we investigated associations between each of the 23 correlates and the target variable number of treatment days using separate univariate linear regression models to test the strength of the individual associations. Outcomes are univariate regression coefficients (βs) that can be interpreted as effect sizes as variables have been standardized. Second, to test the associations between variables when controlling for all other variables, we computed a multiple linear regression analysis. Outcomes are multivariate regression coefficients (βs) that can be interpreted as effect sizes. As classic regression models tend to result in overfitting (especially when the number of predictors is high relative to the number of cases, [45]) and thereby overestimate both the associations between correlates and target variable and the amount of variance that can be explained, we used tenfold cross validation to provide a better estimate in regards to the explanatory power of the regression model. Third, we computed a Least Absolute Shrinkage and Selection Operator (LASSO) regression model (using an α-value of 0.95 and fitting the model with λ = 0.065) to provide an alternative machine learning approach to control for overfitting. LASSO regression introduces a penalization term to the estimation procedure, in which model coefficients are shrunk towards zero [45] – the method thereby allows us to determine a smaller subset of correlates that exhibit the strongest effects or associations [46]. We again estimate the model’s predictive accuracy using tenfold cross validation. Outcomes are regression coefficients (*b*s), controlling for all other correlates in the model. Coefficients that are not shrunk to 0 can thereby be considered to be the most relevant in explaining the variation in the outcome variable. Fourth, we applied a tree-based machine learning algorithm and used random forest models [45] to estimate the relative importance of each correlate in a non-linear setting. Outcomes are variable importance values that can be ranked according to their height (for more information on the four statistical methods, see [45]). The higher the variable importance value of a correlate, the higher its predictive power in the model.

All correlate variables were standardized before computing the statistical models. As the target variable (number of treatment days) is a count variable and therefore may not best be described by a normal distribution, we applied a log-transformation and checked for the distribution of residuals. For descriptive statistics we used IBM SPSS Statistics 27 [47], for all other analyses we used R [48] with the packages glmnet [49], caret [50], grpreg [51], and pROC [52].

## Results

**Fig. 1:**
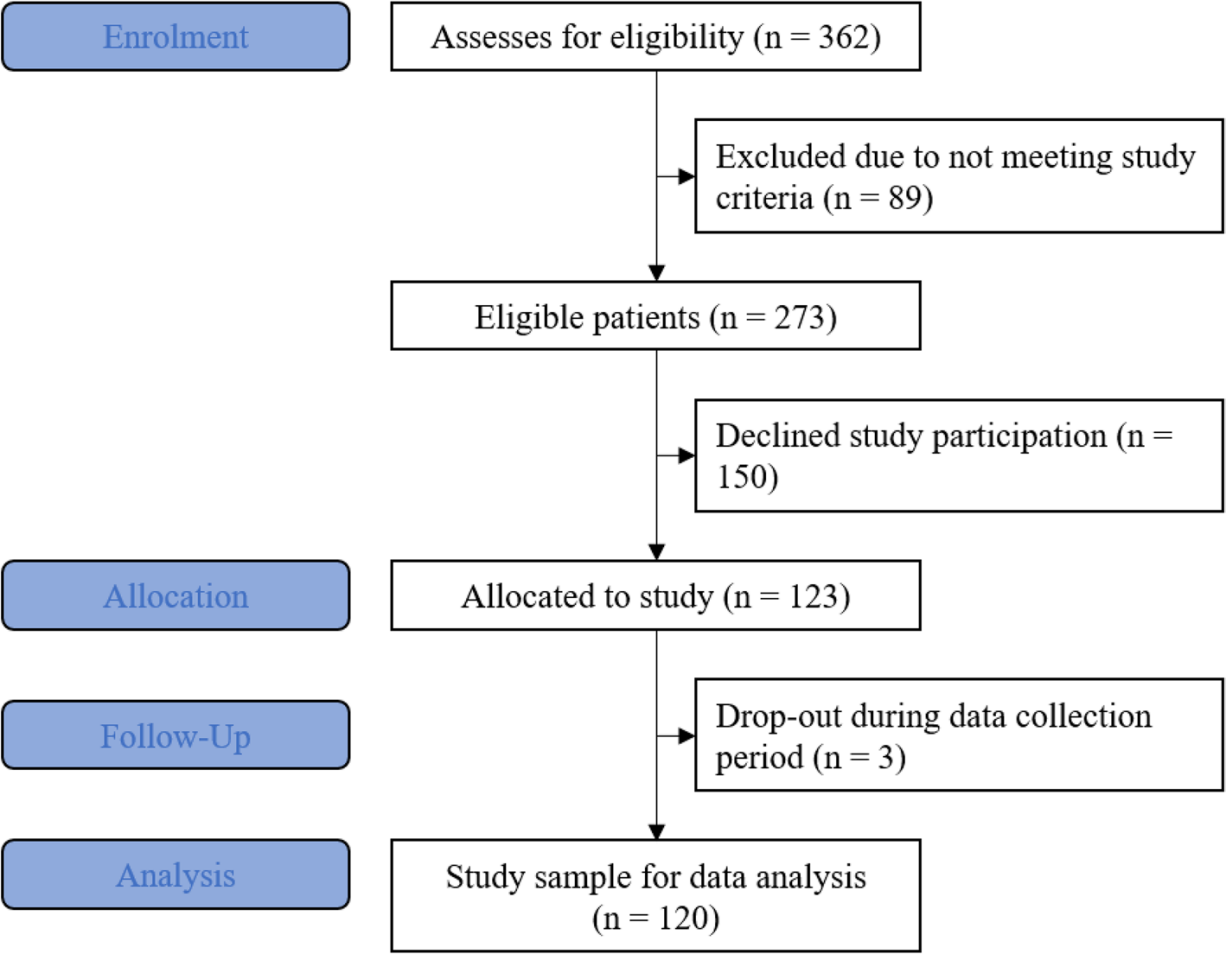
Summary of enrolment process and sample formation using an adapted CONSORT flowchart for survey setups

The sample of this survey consisted of 120 participants (69 male, 51 female, *M*_*age*_ = 39.72, *SD* = 13.03, Range = 18–64), 30 from each of the four different psychiatric wards (see Fig. 1 for more details on the enrolment process).

### Descriptive results

The median number of inpatient treatment days within the last 30 months (excluding the stay during this study) was 6.00. The median number of stays in inpatient psychiatric clinics including the current stay was 2. We further assessed treatment days in day clinics within the last 6 months (*Md* = 0) and the number of outpatient sessions in the last 6 months with a psychiatrist (*Md* = 4) and with a clinical psychologist (*Md* = 0). A detailed overview of the utilization of psychiatric inpatient treatment and related services can be found in Table 1.

**Table 1:** Utilization of psychiatric treatment and related services of the study sample (*n* = 120)

Utilization of services	10% P	25% P	Md	75% P	90% P
Treatment days in inpatient psychiatric clinics (excl. stay during study) within the last 30 months	0.00	0.00	6.00	87.00	178.20
Number of stays in inpatient psychiatric clinics (incl. the current stay)	1.00	1.00	2.00	4.00	6.90
Treatment days in day clinics within the last 6 months	0.00	0.00	0.00	0.00	1.80
Number of outpatient contacts outside of the local clinic context within the last 6 months	0.00	0.00	3.00	13.00	24.00
Number of outpatient contacts within the local clinic context within the last 6 months	0.00	0.00	0.00	3.00	7.00
Number of outpatient contacts within the last 6 months provided by psychiatrists	0.00	1.00	4.00	13.00	24.00
Number of outpatient contacts within the last 6 months provided by psychologists	0.00	0.00	0.00	0.00	5.80
Number of complementary treatments/support within the last 6 months^a^	0.00	0.00	0.00	3.75	83.10
Number of outpatient somatic treatments within the last 6 months	0.00	0.00	0.00	3.00	7.90
Number of complementary nursing home care contacts within the last 6 months	0.00	0.00	0.00	0.00	12.70

Stays in forensic clinics, somatic hospitals and other support (e.g., guardianship) as well as the receipt of psychotherapy in the previous 5 years were assessed, but were outside of the focus of this manuscript and the results are therefore not presented here

*Md* Median, *P* Percentile

^a^Complementary treatment/support may include protected workshops, occupational therapy, contact/advice centres, social psychiatric services, self-help groups

According to the high utilization criteria, 31 participants (25.8%) showed a HU pattern and 89 (74.2%) a NHU pattern. Table 2 summarizes information on the socio-demographic characteristics of participants in general and on participants with a pattern categorized as HU or NHU.

**Table 2:** Summary of sociodemographic characteristics of study participants

		**Overall** **(** ***n*** **= 120)**	**High Utilization pattern** **(** ***n* = 31)**	**Non-high Utilization pattern** **(** ***n* = 89)**
Gender	Male	69 (57.5%)	17 (54.8%)	52 (58.4%)
	Female	51 (42.5%)	14 (45.2%)	37 (41.6%)
Age^a^		39.72 (13.03)	39.48 (13.50)	39.80 (12.94)
Nationality	Swiss	83 (69.2%)	25 (80.6%)	58 (65.2%)
	Other	37 (30.8%)	6 (19.4%)	31 (34.8%)
Educational level	Low level (max. mandatory school)	45 (37.5%)	14 (45.2%)	31 (34.8%)
	Medium level (up to A-levels)	64 (53.3%)	17 (54.8%)	47 (52.8%)
	High level (University degree)	11 (9.2%)	0 (0.0%)	11 (12.4%)
Family status	Not in a relationship	99 (82.5%)	26 (83.9%)	73 (82.0%)
	In a relationship	21 (17.5%)	5 (16.1%)	16 (18.0%)
Living situation	Private living situation	87 (72.5%)	16 (51.6%)	71 (79.8%)
	Assisted living situation	29 (24.2%)	14 (45.2%)	15 (16.9%)
	Other	4 (3.3%)	1 (3.2%)	3 (3.4%)
Working situation	Regular labour market	25 (20.8%)	1 (3.2%)	24 (27.0%)
	Protected labour market	26 (21.7%)	11 (35.5%)	15 (16.9%)
	No job	69 (57.5%)	19 (61.3%)	50 (56.2%)
Receipt of support^b^	Yes	91 (75.8%)	27 (87.1%)	64 (71.9%)
	No	29 (24.2%)	4 (12.9%)	25 (28.1%)
Receipt of disability pension	Yes	65 (54.2%)	22 (71.0%)	43 (48.3%)
	No	55 (45.8%)	9 (29.0%)	46 (51.7%)
Contact with the police within last 6 months	Yes	53 (44.2%)	14 (45.2%)	39 (43.8%)
	No	67 (55.8%)	17 (54.8%)	50 (56.2%)

^a^For this variable the statistical mean value (and standard deviation) is reported

^b^Receipt of support may include, e.g., unemployment benefits, social care, disability pension, or care allowances

Table 3 summarizes the psychiatric characteristics of the study participants. In general, 62 (51.7%) patients had at least one somatic disease (the average number of somatic diagnoses was 1.18, *SD* = 1.73), and 71 (59.2%) had an additional comorbid mental health disorder. Fifty-five (45.8%) had an F2X diagnosis and the average duration of the mental health illness was 44.92 months (*SD* = 74.97).

**Table 3:** Summary of psychiatric characteristics of study participants

		**Overall** **(** ***n* = 120)**	**High Utilization pattern** **(** ***n* = 31)**	**Non-High Utilization pattern** **(** ***n* = 89)**
Somatic diagnosis	Yes	62 (51.7%)	17 (54.8%)	45 (50.6%)
	No	58 (48.3%)	14 (45.2%)	44 (49.4%)
Number of somatic diagnoses^a^		1.18, (1.73)	1.26, (2.05)	1.15, (1.61)
Psychiatric diagnosis (ICD-10)	F2X	55 (45.8%)	19 (61.3%)	36 (40.4%)
	F3X	40 (33.3%)	8 (25.8%)	32 (36.0%)
	F4X	8 (6.7%)	1 (3.2%)	7 (7.9%)
	F6X	6 (5.0%)	3 (9.7%)	3 (3.4%)
	F8X	2 (1.7%)	0 (0.0%)	2 (2.2%)
	F9X	1 (0.8%)	0 (0.0%)	1 (1.1%)
	Missing	8 (6.7%)	0 (0.0%)	8 (9.0%)
Psych. Comorbidity	Yes	71 (59.2%)	20 (64.5%)	51 (57.3%)
	No	49 (40.8%)	11 (35.5%)	38 (42.7%)
Addiction as comorbidity	Yes	48 (40.0%)	12 (38.7%)	36 (40.4%)
	No	72 (60.0%)	19 (61.3%)	53 (59.6%)
Duration of psychiatric illness in months^a^		44.92, (74.97) (*n* = 79)	71.40, (68.91) (*n* = 20)	35.95, (75.36) (*n* = 59)
Attempts of suicide in medical history	Yes	26 (21.7%)	10 (32.3%)	16 (18.0%)
	No	94 (78.3%)	21 (67.7%)	73 (82.0%)
BPRS Sum-Score^a^	Total^a^	44.11, (11.25)	47.16, (12.31)	43.04, (10.73)
	Anxiety depression^a^	14.77, (4.92)	16.06, (5.10)	14.31, (4.80)
	Anergia^a^	6.31, (2.69)	7.06, (2.87)	6.04, (2.59)
	Thought disturbance^a^	9.15, (4.89)	10.58, (5.82)	8.65, (4.46)
	Activation^a^	6.42, (2.82)	6.45, (2.89)	6.40, (2.82)
	Hostile suspiciousness^a^	7.47, (4.06)	7.00, (3.99)	7.63, (4.10)
GAF-Score^a^		30.37, (11.34)	25.94, (10.32)	31.91, (11.32)
Assignment to clinic	involuntary	23 (19.2%)	4 (12.9%)	19 (21.3%)
	voluntary	95 (79.2%)	25 (80.6%)	70 (78.7%)
	Missing	2 (1.7%)	2 (6.5%)	0 (0.0%)

*BPRS* Brief Psychiatric Rating Scale, *GAF* Global Assessment of Functioning

^a^For these variables the statistical mean values (and standard deviation) are reported

Table 4 summarizes the psychological characteristics and unmet needs of the study participants. Overall, participants reported *M* = 3.41 unmet needs (*SD* = 2.69). Participants with a pattern categorized as HU reported on average 4.29 unmet needs. When looking at the different needs assessed in the study, differences of ≥ 10% points were found between participants with a pattern categorized as HU versus NHU for needs regarding the household (25.8% vs. 5.6%), daily activities (38.7% vs. 28.1%), psychotic symptoms (29.0% vs. 10.1%), social contacts (48.4% vs. 37.1%) and sexuality (45.2% vs. 27.0%; see Table 4).

**Table 4:** Summary of psychological characteristics and unmet needs of study participants

		**Overall** **(** ***n* = 120)**	**High Utilization pattern** **(** ***n*** **= 31)**	**Non-high Utilization pattern** **(** ***n*** **= 89)**
DAI-Score^a^	Total^a^	1.23, (4.83)	0.13, (4.95)	1.62, (4.76)
	Subjective positive response^a^	0.34, (2.23)	0.23, (2.17)	0.38, (2.26)
	Subjective negative response^a^	−0.05, (2.17)	−0.87, (2.06)	0.24, (2.14)
	Attitude to medication^a^	0.94, (2.21)	0.77, (2.17)	1.00, (2.24)
CANSAS^a^	Number of unmet needs^a^	3.41, (2.69)	4.29, (3.27)	3.10, (2.41)
Needs present and not met in regards to	Living situation	7 (5.8%)	1 (3.2%)	6 (6.7%)
	Diet	8 (6.7%)	4 (12.9%)	4 (4.5%)
	Household	13 (10.8%)	8 (25.8%)	5 (5.6%)
	Personal hygiene	10 (8.3%)	4 (12.9%)	6 (6.7%)
	Daily activities	37 (30.8%)	12 (38.7%)	25 (28.1%)
	Physical health	20 (16.7%)	6 (19.4%)	14 (15.7%)
	Psychotic symptoms	18 (15.0%)	9 (29.0%)	9 (10.1%)
	Info. about illness/treatment	9 (7.5%)	1 (3.2%)	8 (9.0%)
	Mental pressure	62 (51.7%)	16 (51.6%)	46 (51.7%)
	Self-endangerment	30 (25.0%)	10 (32.3%)	20 (22.5%)
	External danger	7 (5.8%)	3 (9.7%)	4 (4.5%)
	Alcohol	4 (3.3%)	1 (3.2%)	3 (3.4%)
	Illegal drugs	7 (5.8%)	4 (12.9%)	3 (3.4%)
	Social contacts	48 (40.0%)	15 (48.4%)	33 (37.1%)
	Relationships	42 (35.0%)	10 (32.3%)	32 (36.0%)
	Sexuality	38 (31.7%)	14 (45.2%)	24 (27.0%)
	Care of children	6 (5.0%)	2 (6.5%)	4 (4.5%)
	Knowledge in reading, writing, calculating	1 (0.8%)	0 (0.0%)	1 (1.1%)
	Phone	3 (2.5%)	1 (3.2%)	2 (2.2%)
	Transportation	6 (5.0%)	2 (6.5%)	4 (4.5%)
	Money	22 (18.3%)	6 (19.4%)	16 (18.0%)
	Social support/benefits	11 (9.2%)	4 (12.9%)	7 (7.9%)

*CANSAS* Camberwell Assessment of Need Short Appraisal, *DAI* Drug Attitude Inventory

^a^For these variables the statistical mean values (and standard deviation) are reported

### Inferential statistics

Results of all models regarding correlates of the number of treatment days are presented in Table 5.

**Table 5:** Summary of results from various inferential analyses examining at psychiatric inpatient treatment utilization as continuous outcome variable

**Characteristic**	**Coding**	**Univariate Regression Outcomes**	**Multivariate Regression Outcomes**	**LASSO Regression Outcomes**	**Random Forest Outcomes** ^a^
Intercept			β = −0.00 (0.08), *t* = 0.00, *p* = 1.000		
Gender	0 = male 1 = female	β = 0.18 (0.09), *t* = 1.94, *p* = .055	β = 0.24 (0.10), *t* = 2.33, *p* = .022	*b* = 0.11	2.85
Age		β = −0.01 (0.09), *t* = −0.14, *p* = .886	β = −0.04 (0.10), *t* = −0.39, *p* = .701	*b* = 0.00	6.80
Nationality	0 = Swiss 1 = other	β = −0.16 (0.09), *t* = −1.75, *p* = .084	β = −0.05 (0.10), *t* = −0.49, *p* = .623	*b* = −0.02	1.94
Family status	0 = no relat 1 = in a relat	β = −0.12 (0.09), *t* = −1.36, *p* = .177	β = −0.07 (0.09), *t* = −0.76, *p* = .451	*b* = −0.02	1.46
Educational level	0 = low 1 = medium 2 = high	β = −0.15 (0.09), *t* = −1.60, *p* = .112	β = 0.06 (0.10), *t* = −0.57, *p* = .570	*b* = −0.02	2.98
Working situation	0 = no job 1 = prot. lab. market 2 = reg. lab. market	β = −0.13 (0.09), *t* = −1.46, *p* = .147	β = 0.04 (0.10), *t* = 0.37, *p* = .714	*b* = 0.00	4.01
Living situation	0 = other 1 = supported living	β = 0.27 (0.09), *t* = 3.10, *p* = .002	β = 0.16 (0.09), *t* = 1.68, *p* = .097	*b* = 0.14	3.88
Receipt of disability pension	0 = no 1 = yes	β = 0.20 (0.09), *t* = 2.22, *p* = .028	β = 0.13 (0.12), *t* = 1.10, *p* = .274	*b* = 0.05	2.75
F2X-Diagnosis	0 = no 1 = yes	β = 0.23 (0.09), *t* = 2.56, *p* = .012	β = 0.09 (0.16), *t* = 0.55, *p* = .585	*b* = 0.05	2.62
F30/F31-Diagnosis	0 = no 1 = yes	β = −0.01 (0.09), *t* = −0.07, *p* = .943	β = −0.00 (0.14), *t* = −0.03, *p* = .979	*b* = 0.00	1.41
F32/F33-Diagnosis	0 = no 1 = yes	β = −0.24 (0.09), *t* = −2.71, *p* = .008	β = −0.07 (0.13), *t* = −0.52, *p* = .606	*b* = −0.08	2.57
Mental health comorbidity	0 = no 1 = yes	β = 0.18 (0.09), *t* = 2.04, *p* = .044	β = 0.09 (0.13), *t* = 0.70, *p* = .487	*b* = 0.06	2.43
Addiction as comorbidity	0 = no 1 = yes	β = 0.17 (0.09), *t* = 1.87, *p* = .064	β = 0.10 (0.13), *t* = 0.83, *p* = .411	*b* = 0.07	2.36
BPRS—Anxiety depression		β = 0.07 (0.09), *t* = 0.74, *p* = .458	β = −0.11 (0.12), *t* = −0.93, *p* = .356	*b* = 0.00	6.31
BPRS—Anergia		β = 0.16 (0.09), *t* = 1.75, *p* = .083	β = 0.04 (0.11), *t* = 0.42, *p* = .675	*b* = 0.00	5.71
BPRS—Thought disturbance		β = 0.22 (0.09), *t* = 2.46, *p* = .016	β = 0.06 (0.14), *t* = 0.41, *p* = .682	*b* = 0.01	6.38
BPRS—Activation		β = 0.09 (0.09), *t* = 1.00, *p* = .318	β = 0.02 (0.11), *t* = 0.21, *p* = .834	*b* = 0.00	5.11
BPRS—Hostile suspiciousness		β = −0.00 (0.09), *t* = −0.01, *p* = .988	β = −0.20 (0.11), *t* = −1.78, *p* = .078	*b* = −0.06	6.02
GAF Score		β = −0.29 (0.09), *t* = −3.23, *p* = .002	β = −0.16 (0.13), *t* = −1.24, *p* = .219	*b* = −0.13	8.20
DAI: Subjective positive response		β = −0.02 (0.09), *t* = −0.16, *p* = .870	β = −0.11 (0.11), *t* = −1.01, *p* = .316	*b* = 0.00	3.23
DAI: Subjective negative response		β = −0.19 (0.09), *t* = −2.16, *p* = .033	β = −0.14 (0.10), *t* = −1.44, *p* = .154	*b* = −0.08	4.70
DAI: Attitude to medication		β = −0.01 (0.09), *t* = −0.15, *p* = .883	β = 0.08 (0.11), *t* = 0.70, *p* = .484	*b* = 0.00	3.64
Unmet needs		β = 0.22 (0.09), *t* = 2.39, *p* = .018	β = 0.24 (0.11), *t* = 2.14, *p* = .035	*b* = 0.12	7.14
Overall Model Fit		-	*R* ^*2*^ = .34 *Adj. R* ^*2*^ = .18		
Fit after Cross-Validation		-	*R* ^*2*^ = .16 *RMSE* = 1.02	*R* ^*2*^ = .16 *RMSE* = 0.97	*R* ^*2*^ = .22 *RMSE* = 0.94

Data from all participants was included in these analyses

^a^Variable importance values are reported

For the demographic variables, the results from the univariate regression indicated a positive association between the number of treatment days during the last 30 months and both the living situation (an assisted living situation compared to a private or other living situation), β = 0.27, as well as being the recipient of a disability pension (the receipt being positively associated with more treatment days), β = 0.20, respectively. Furthermore, significant positive associations were found between the number of treatment days, a current F2X diagnosis (β = 0.23), and the current presence of mental health comorbidities (β = 0.18). Further associations were found between the number of treatment days and a current F32/F33 diagnosis (β = −0.24), the amount of thought disturbance on the specific subscale of the BPRS (β = 0.22, with higher values being associated with more treatment days), and the GAF-score (β = −0.29, with a higher functioning level being associated with fewer treatment days). On the level of psychological attributes, we found negative associations between the number of treatment days and current subjective negative responses to medication (β = −0.19, higher values on this scale indicate less negative attitudes) and positive associations between number of treatment days and the current number of unmet needs (β = 0.22).

Applying a multiple regression model and testing associations in the context of all other correlates, our results demonstrate that only age and number of unmet needs remained correlates for the number of treatment days. This result was complemented by the LASSO regression model. The LASSO model identified living situation, the GAF-score and the number of unmet needs as three relevant correlates. The weights for the predictors age, working situation, anxiety-depression (BPRS), activation (BPRS), subjective positive responses to medication, attitude to mediation, and presence of a current F30/F31 diagnosis were all shrunk to 0.00 by the model. The Random Forest model, in turn, identified the GAF-score, unmet needs, and age as the three most important statistical correlates of number of treatment days.

The multiple regression model explained 34% of variance (*R* ^*2*^ = 0.34, *f* ^*2*^ = 0.52, cf., Table 5). Looking at values after cross validation, the models explained a range of 16 to 22% of variance (*R* ^*2*^_*max*_ = 0.22 for the Random Forest model).

## Discussion

This study gives detailed information on inpatient utilization of patients admitted to a psychiatric hospital in a catchment area of Northern Switzerland. It provides an analysis of potential correlates of this utilization by applying different statistical models including machine learning analyses. Although our analyses explain a moderate part of the variation in the number of treatment days, the only variable that turned out to be a meaningful correlate of the number of treatment days across all statistical models applied was the number of unmet needs. Unmet needs may therefore represent a promising variable for future research designs that aim to understand and investigate strategies to prevent or reduce unnecessary psychiatric hospitalizations.

In our study, many of the unmet needs reported by participants point to social factors: Whereas only 3–7% of participants reported unmet needs in the domains of living situation, diet, phone and transportation, 40% reported unmet needs with regard to social contacts (48% HU; 37% NHU), 35% with insufficient relationships (HU 32%; NHU 36%) and 32% with regard to sexuality (HU 45%; NHU 27%). Furthermore, 31% (HU 39%; NHU 28%) of participants described unmet needs when it comes to daily activities. These findings are corroborated by an investigation of Freeman and colleagues [53], who asked patients with a schizophrenia diagnosis to choose intervention targets from a list but also allowed them to name additional targets. Almost 60% of their participants chose increasing activities as an intervention target from the list and among the additionally named targets, social connectedness and practical support was dominant [53]. Other work also highlights that social isolation and feeling lonely were the most highly endorsed unmet needs people with severe mental illness report [54]. Among the potential reasons for unmet social needs are impaired social skills, lack of opportunities to participate in social activities, and social stigma linked to mental illness.

The importance of unmet needs is in line with previous research looking at quality of life in general [55, 56] but also specifically when it comes to mental health treatment [57, 58]. While diagnoses and previous hospitalizations are linked to future hospitalizations (see [59]), self-reported ongoing struggles with symptoms [59] and the patient’s view on unmet needs could be important facets when aiming to understand the search for and utilization of psychiatric care.

The type of self-reported unmet needs are a potentially clinically relevant factor as they are modifiable and can be addressed and targeted through need-centred clinical interventions [60]. Pathare and colleagues [61] also describe this idea when introducing the term *care gap* (in contrast to a treatment gap which covers mostly medical treatment). The care gap highlights psychosocial interventions as important key component [62].

Psychosocial interventions, in turn, might be better implemented in community mental health services outside of hospitals [63], which could focus more on the specific unmet needs in the (social) context in which they occur (which is outside of the hospital). Tailored treatment programmes that may better target unmet needs of patients in their communities (versus traditional care based on low intensive outpatient or, in case of any crisis, high intensive hospital-centred interventions) are outreach programmes such as home treatment during a mental health crisis [64–66] or assertive community treatment [67]. The implementation of these outreach services is evidence-based and recommended in a variety of treatment guidelines (see, e.g., [68]). In Switzerland, however, home treatment and other outreach programs are not (yet) widely available in routine care settings due to the absence of a legal framework and established funding through basic health insurance [69]. This causes implementation challenges. Some programs have been implemented and others are currently being introduced, but they are primarily being offered as pilot projects [70] with different financial models.

If our study findings are replicated, future research could use randomized controlled trials within specific health systems such as the Swiss one to assess whether specific measures addressing self-reported unmet needs can reduce hospital use. First studies have shown that mental health outreach programmes such as assertive community treatment (cf., [67]) may have the potential to reduce patients’ unmet needs while providing the support within patients’ communities [60].

As a secondary finding, our descriptive analyses indicate that the patients included in this study only rarely utilized outpatient psychotherapy by a clinical psychologist in the last 6 months. This may indicate an undersupply of outpatient psychotherapy for people with severe mental illness and is in line with prior findings in the same catchment area [71].

### Limitations

Given the retrospective design of the study, some limitations apply. For the inferential statistical analysis, we labeled the utilization behavior as the target variable and the psychiatric, psychological, and sociodemographic variables as correlates. This, however, is an application of statistical terms and does not reflect any causal inferences that can be derived from the results. All variables were assessed at the same time during our study, and utilization behavior over 30 months was investigated in retrospect. This may limit the amount of variance that can be explained with statistical predictor variables that might have changed during the timeframe when the behavior of seeking inpatient treatment occurred (e.g., attitudes towards medication, living situation). Longitudinal research may be conducted to examine if the correlates chosen in the study might also be predictive for future utilization of inpatient treatment.

While electronic patient records allowed us to assess precise data on inpatient treatment within our clinic, information on additional treatment outside of our clinic could not be drawn from our clinical information system. Twenty-two patients reported having additional inpatient treatment episodes in other clinics in the interview and provided estimates for dates and duration, which were integrated into the data on utilization behavior. Self-report bias with potentially missing data may, in turn, introduce additional error variance into the statistical models. Moreover, psychiatric inpatient care is a complex phenomenon that may involve many different patient and environmental factors that would need to be considered. While we offer an investigation of 23 variables, many others could still be of interest, which may explain the rather low number of explained variance. One example could be patients’ subjective perceptions about their illness. Previous work has shown that illness perceptions are associated with patients’ needs and with their health outcomes [72]. Another example could be self-stigmatization and anticipated or experiences stigmatization of mental health care personnel (e.g., [73]). Future studies could include such variables in the research and analysis plan to potentially increase the variance that can be explained in inpatient use.

We, further, cannot rule out the possibility of selection biases, as we were unable to compare study participants with non-participants treated in the relevant wards during the study period. If present, such a bias would pose a limitation in regards to the external validity of our findings [74]. Future research may aim to replicate our findings, ideally with larger sample sizes, different scales, and potentially in a different health setting. While we believe a precise description of a sample of 120 patients with severe illnesses is important to learn more about potential target groups in acute psychiatric settings, a larger sample size would be desirable to increase statistical power. Furthermore, we found that the reliability of the DAI-subscales was low, which means that given measurement uncertainty, results should be interpreted with caution and future research may identify other suitable scales to assess attitudes towards medication.

## Conclusion

Taken together, this study joins a body of research examining the utilization of inpatient psychiatric treatment with the goal of better understanding high utilization patterns and tailoring treatments to the specific needs of the affected people. Results should feed into efforts to improve mental health care services for this vulnerable patient population. Given further replication, this study identifies unmet patient needs as a potential variable of interest that needs to be addressed.

## Data Availability

The data is not publicly available but an anonymized version of the here presented variables can be shared upon request to any qualified researcher.

## Appendix

### Appendix B: Additional information on study materials and procedure

The patients and interviewer completed the BPRS and GAF scale, and the CSSRI questions. The CSSRI was complemented by two questions inquiring about outpatient treatment provided by a psychologist and about outpatient psychotherapy during the last 5 years. Both questions are part of a different research project and are not the focus of this manuscript. Participants completed the CANSAS questions (CANSAS-P as it focused on the patients’ perspective) and provided answers on the DAI-10 scale. Patients were then asked three open questions about their opinion on reasons for their current stay in the psychiatric hospital, which reasons were most impactful, and what could have prevented the current stay. Participants could then ask questions and were thanked for their participation in the study.

Next to the interview itself, participants were also asked whether the researchers could contact a close family member or friend by phone for a few follow-up questions [40]. Sixty participants agreed to this procedure, however, only 41 contact persons could be reached via phone. We also conducted additional qualitative interviews with 23 participants whose behavioral pattern regarding clinic stays could be described as high utilization. Both follow-up projects were, however, not the main focus of the study and are therefore not reported in this manuscript.
